# Supplementary material for: Creating speech zones with self-distributing acoustic swarms
Source: Nat Commun. 2023 Sep 21;14:5684. doi: 10.1038/s41467-023-40869-8 (PMC10514314; doi:10.1038/s41467-023-40869-8)
Supplement: Supplementary file 1 — Supplementary Information [file 41467_2023_40869_MOESM1_ESM.pdf]

Creating speech zones with self-distributing acoustic swarms

Supplementary Text

Robot hardware. The robots are programmed using a Micro-USB connector. A plastic wheel is slotted through each of the motor shafts, and a rubber o-ring is fitted around the wheel to increase friction. The robot base, wheels and motor holder are 3D printed using a Formlabs Form 3+ with black resin. The base station is 3D printed using an Ultimaker 3 with PLA filament. The photointerruptors are mounted onto a set of angled cantilevers to the front of the robot. Two photointerruptors drive a current through a resistor to obtain a voltage which we digitize using a dual-channel comparator (LM293QT), with a threshold voltage we set using a voltage divider. The photointerruptors can be turned off in software using GPIO through a transistor switch. When the photointerruptors sense a state switch, we read the signal after 10 ms to ensure that the signal has settled.

Fig. S9 plots the output of the photointerruptor for different table colors. To detect collisions, an accelerometer sampling at 1 kHz is used to look for sudden spikes. Specifically, collisions trigger a large variation in the x- and y-components of the accelerometer readings, a_x and a_y respectively. So, we compare $||a_x^2 + a_y^2||$ to a pre-set threshold. When robots are stationary in Fig. 3A, and collisions are expected from other moving robots, we monitor for changes in the accelerometer magnitude and compare it to a threshold of 5 m s^{-2}. When the robot is moving, factors such as surface roughness may contribute to erroneous spikes in the accelerometer measurements. So, to perform collision detection for moving robots, we use absolute accelerometer readings, as opposed to changes, and use a higher threshold of 17 m s^{-2}.

The robots use the gyroscope to move in a straight line. The right and left motors are driven using a separate PWM signal, whose duty cycles, c_R and c_L, are varied to increase or decrease the motor rotation speed. We control the forward motion by changing these duty cycles with a magnitude m and ratio β such that $c_R = 2m\beta$ and $c_L = 2m(1-\beta)$. At start up, we set $\beta = \frac{1}{2}$ and during gyro-controlled forward motion, we use a proportional controller to stabilize the yaw rate to zero by changing β. The controller computes a value to add to β from the current yaw rate. The proportional term coefficient is set to 0.025 s deg^{-1}, and β is clamped between 0.25 and 0.75.

During controlled forward motion, we also use the value of β to detect collisions. Specifically, collisions on the side of the robot cause the robot to naturally turn to one side. The gyroscope control loop causes the value of β to veer to an extreme value to correct for this turn. So, during a controlled forward motion, if the value of β is not in the range $[0.35, 0.65]$, the robots assume that a collision has occurred.

The robots rotate in place by driving both motors with the same duty cycle in opposite directions. To turn to a target rotation, a proportional controller varies this duty cycle so that the current rotation estimate gets close to the target rotation using a proportional coefficient of 0.12 % deg^{-1}, and clamping the duty cycle between 15% and 22%. Finally, the direction of rotation is given by the sign of the rotation error.

Motion control. If the robot wants to move from a position $\mathbf{P_0} = [x_0, y_0]$ with orientation θ_0 to a milestone at $\mathbf{P_M} = [x_M, y_M]$, it first rotates by $\theta_r = \text{atan2}\ (y_M - y_0, x_M - x_0) - \theta_0$ and moves forward. The target duty cycle is set based on the distance to the target position using a proportional-integral controller, where the proportional and integral term coefficients are set to 1.98 % cm^{-1} and

0.22 % cm^{-1} s^{-1} respectively. The controller-determined duty cycles values are clamped between 14% and 18%. A milestone is considered reached if the line segment joining its current and previous position estimates is within 2 cm from the milestone.

Pre-entry maneuver and docking. To dock into the base, the robot has to enter at close to 90° to avoid hitting the sides of the entrance. During navigation, since the gyroscope drift accumulates, entering at the correct angle is challenging. To accurately estimate its orientation, the robot turns around by 180° and moves away from the base in a straight line for 1.5 s. The robot transmits acoustic chirps every 200 ms and uses linear regression to estimate the angle of motion with respect to its starting position. It then uses this to estimate and correct any orientation error. It then travels back to position itself in a region around the center of the ramp entrance. Finally, it rotates to enter the ramp at 90°.

After entering the base, the robot needs to dock with the grooves on the track. To achieve this, the robot follows a sequence of steps: 1) The robot goes up the entry ramp to enter the base. 2) It rotates away from the base to 270°, and moves backward to touch the back of the entry ramp. 3) It rotates towards the grooves to 0° of the base, and fine tunes its rotation so that the photointerruptors detect both black markers on the tracks in front of the entry ramp. 4) The robot slowly moves forward until the photointerruptors detect the white surface of the platform. 5) The robot shakes by rotating quickly in both directions for 800 ms to slot its wheels in the grooves. 6) Finally, it moves forward until it detects the first position marker.

Note that since rotation tasks are expected to be short, if a robot doesn't finish rotation within some set amount of time, the robot increases the rotation speed by a fixed amount every second until it reaches the target rotation. This is evident during docking, when the robot may need to ramp up its speed because it may get caught in the tape or grooves. To prevent the robot getting stuck during docking, we ramp up the rotation and forward motion speeds by increasing the duty cycle by 10-20% every second. Further, when the robot is in the base, the robots do not use the gyro to control their movement since the grooves are used to guide and rotate the robots.

Audio recording and compression. The microphones are interfaced with Nordic's pulse density modulation (PDM) driver. We use direct memory access (DMA) to asynchronously convert the PDM measurements to pulsed coded modulation (PCM) values 20 ms at a time. Since the driver does not support 48 kHz sampling, we sample audio at 50 kHz and downsample to 48 kHz using linear interpolation. Then, we compress the samples 20 ms at a time. To maximize throughput, we buffer encoded samples and transmit 240 bytes at a time, which corresponds to around 60 ms of compressed audio at 32 kbps per packet. For audio streaming, each robot records from a single microphone. The robots stream audio recordings to an off-the-shelf Nordic nRF52840 development kit, which relays the BLE packets over USB to a host computer to process the results.

Time synchronization. Each robot keeps track of a free-running timer T that overflows every 50 ms and a counter C that increments when the timer overflows. We designate a robot as the Time Sync Server (TSS) and use the Nordic TimeSlotAPI to periodically broadcast time sync packets with TSS timer T_{TSS} and counter C_{TSS}. All other robots are designated as Time Sync Clients (TSC) and continuously listen to time sync packets. Every time a TSC receives a packet, it updates its T and

C values with those of the TSS. We initiate time-sensitive tasks such as recording audio and playing chirp signals by scheduling them before designating the TSS. Specifically, if a task is scheduled at some interval i, then when the value of C reaches $i-1$, the controller arms a Programmable Peripheral Interconnect to start this task the next time T overflows. Additionally, since the robot peripherals use the system clock, we need to compensate for the clock drift between the system and the server clocks. For recording tasks, the robot saves the server and system clocks at the start of the recording. When the system clock drifts more than the equivalent of one sample, we compensate by adding or dropping an audio sample in our recording.

Sensor coordination on robots. Sensor coordination among microphones, accelerators, and gyroscopes plays an important role in robot localization and navigation. To illustrate the superiority of sensor coordination, we control the robot to move in a curve. We place an overhead camera to capture the movement of the robot and use the optical flow algorithm to extract a trajectory for the robot's movement (see the blue curve in Fig. S10). Then we record the IMU and acoustic localization data and perform localization offline. In Fig. S10, the green curve is the trajectory inferred from just the IMU data (accelerators and gyroscopes). In the beginning, the path is accurate and as time goes on, the drifting becomes larger and larger. The black cross symbol is the localization result from the acoustic data and the red curve is the path from our fuse algorithm of IMU and acoustic data. As we can see, acoustic localization can help avoid the error accumulation from IMU data. On the other hand, even though acoustic localization can provide accurate localization without drifting, it can only provide some discrete points without rotation estimation. Hence, the accelerators and gyroscopes can help track the positions and orientations of robots.

Surface Occupancy Ratio. Intuitively, this measures how wide an array appears at a given angle versus how wide it can appear to be for a given shape. This metric can be used to evaluate how effectively the swarms utilize the desk space. An illustration of this metric is shown in Figure. S11. Formally, let $\mathbf{p_1}, \mathbf{p_2}, \ldots, \mathbf{p_M}$ be the positions of robots and $\mathbf{v_1}, \mathbf{v_2} \ldots, \mathbf{v_N}$ be the vertices of the desk. Then, the surface occupancy ratio at a particular angle, θ, is formulated as follows:

$$\frac{\mathrm{d}(\theta)}{\mathrm{d}_{\max}(\theta)} = \frac{\max_{i,j}(\mathbf{p_i} - \mathbf{p_j})[\sin(\theta), -\cos(\theta)]^T}{\max_{i,j}(\mathbf{v_i} - \mathbf{v_j})[\sin(\theta), -\cos(\theta)]^T} \tag{1}$$

Output and Ground-truth matching for evaluation. Our pipeline will output the positions and separated audio of several candidates. To calculate the evaluation metrics including precision, recall, localization error and SISDRi, we need to match the outputs with the ground-truth. The matching process is as follows: we iterate over all possible permutations between the outputs and ground-truth. For each permutation, we check whether the output and its corresponding ground-truth are within 1m and their signals' SISDR is larger than -15dB. If it is satisfied, we regard this output and ground-truth as a matched pair. Then for this permutation, we sum up the localization error and SISDR value of the matched pair. Finally, we select the permutation with highest matched number, lowest localization error, and highest SISDR. In this optimal permutation, we regard the unmatched outputs as the false positive and the unmatched ground-truth as the false negative. We then calculate and report the localization error and SISDRi metric on the matched pair.

Datasets. We trained our networks by using synthetic datasets as well as the data that we collected in both an anechoic chamber and real-world environments. The synthetic data were generated using PyRoomAcoustics (*1*) by simulating several 3-dimensional rooms, where the room lengths and widths were randomly sampled from a uniform distribution of 6–8 m while the heights were sampled from a uniform distribution of 2–2.5 m. We assumed that 7 microphones were placed near one of the walls at a height of 2 cm above the floor as our robots were 2-cm high. One microphone was placed at the center of the microphone array, the remaining 6 microphones were spread with approximately equal angular spacing as with our dispersal algorithm. The angle along which each microphone disperses was perturbed by a random angular displacement uniformly distributed between $\pm 6°$. Each robot was placed near the edge of the desk along the dispersal angle, and its X- and Y-coordinates were randomly perturbed by up to 8 cm. The length and width of the desk on which the microphones are placed were uniformly sampled between 1.2–2 m and 0.6–1.2 m respectively. We randomly placed 2 to 5 speakers in a 6×4.5 m region around the desk and select the height difference between speaker and microphones are uniformly sampled from 0.1–0.8 m. Each speaker emitted an audio segment taken from the VCTK corpus. It contains speech data from 109 native English speakers with various accents. The audio segments were chosen such that the speakers in the same room were different. We removed the leading and trailing silence from each speaker signal, and either segmented it or padded it with zeros to obtain a signal with a target duration. We simulated the room reverberation by randomly setting the wall absorption coefficient value between 0.1 and 0.99. The reverberated signal of each speaker at the first, central microphone position (i.e., the reference channel) was used as the ground-truth signal. Finally, to model the microphone position errors, we randomly time-shifted each microphone channel (not including the reference channel) by a small number of samples in either direction. For the results shown in Fig. 6, our model was trained on the synthetic data. Each synthetically generated sample was 3 s long. Overall, the synthetic dataset consisted of 8000 training samples, 500 validation samples and 1000 testing samples. No speaker was chosen in more than one of these datasets.

To ensure that our model generalizes to real world data, we also augmented the synthetic dataset with samples collected from our robot swarm using portable speakers as audio sources. The data were recorded in both an anechoic chamber and reverberant real-world environments. In the anechoic chamber, we collected data by placing the speakers in various positions between 1 and 5 m from the center of the acoustic swarm, with heights ranging between 0.4 and 1.2 m above the surface of the acoustic swarm. The swarm is placed on a table and microphone positions were obtained using the swarm's 2D localization algorithm. We attached another robot microphone and speaker circuit to the speaker diaphragm and use this circuit to emit a synchronized chirp at this position. We recorded this chirp with the acoustic swarm and used it to compute the ground truth speaker position. Each speaker played a 15-second audio sample from the VCTK dataset. For each microphone placement, we recorded a total of 10 speakers placed at different locations. To generate mixtures from anechoic data, we randomly chose 3 to 5 speakers from a single microphone placement, and obtained a mixture signal by summing the signals from all speakers at each channel. The recordings at each channel included noise from HVAC systems as well as thermal noise from the microphones. Since neither of these signals were produced by the speakers, we cannot use the recording from the reference channel

directly as the ground truth. Instead, we denoise the received signal from each speaker and used this denoised signal as the ground truth for the joint separation network. Specifically, we used a Python non-stationary noise reduction library (*2*) to denoise the single speaker recording, given a 300 ms audio segment before the speaker played its sound. In total, we collected data over 6 different microphone placements, from which we generate 6,000 mixture samples, all of which are used for training.

Finally, we also collected data from real world environments. We employed a similar data collection scheme as with the anechoic chamber data collection. We conducted the data collection in 10 different environments. In each environment, we placed the robot microphones in a different geometry, and collected data from 6 to 7 speakers, depending on the room size. We placed our robots on tables with lengths varying between 1 and 2.5 m, and widths ranging between 0.5 and 0.8 m. The speakers were placed in different positions, and at heights varying from 0.3 m to 1.2 m from the surface of the robot swarm microphone array. Each speaker played 10–15 seconds of randomly chosen VCTK audio clips. The microphone and speaker positions were obtained in the same way as in the anechoic chamber. To generate a mixture sample from these recordings, we randomly chose 3 to 5 speakers and mixed them. We also denoised the recorded signals from isolated speakers to obtain the ground truth signals. We generated 6000, 1000, and 500 samples to use for the training, testing and validation sets, respectively. We used 4 rooms for training (Fig. S12A-D), 1 room for validation (Fig. S12E), and 5 rooms for testing (Fig. 5A-C and Fig. S6). There are no overlapping rooms across these datasets. For the anechoic and real world collected samples only, speakers may overlap between the train, evaluation and test sets. To report the results on tables with objects on them, we run our evaluation on 500 mixtures. We don't train or validate the models on any such mixtures.

We also estimated the RT60 values for the rooms used in our evaluation. This is the time it takes for sound to attenuate by 60dB. We measure this value by placing our robots in the same configuration as we did during data collection, and begin collecting 10 seconds of audio from all robots. During these 10 seconds, we pop a balloon to obtain an approximate room impulse response at every robot. Then, we find the peak value in the recorded signals at each robot and consider the impulse response to start at this peak value. Since the rooms we measure are noisy, we use the PyRoomAcoustics measure_rt60 function to first measure the time for the sound to decay by 20 dB, and then extrapolate to obtain RT60. We use all robots to obtain 7 different estimates for RT60 and report the average and standard deviation in Table S3.

We augmented our synthetic data with both the anechoic and real-world data described above. Specifically, we reduced the number of synthetic mixtures to 6000, which we use for training only. We used this combination of synthetic, anechoic and reverberant datasets to train our models to generate the results in Supplementary Fig. 5 and Figs. 8 and 9.

Training. The localization by separation network was trained by sampling points in a room and setting the target output to be the signal from the closest speaker within a hypercube whose width is randomly sampled from $\{2, 4\}$. If no such speaker exists, then the target output is a zero signal with the same duration as the input. During training, we chose points that give zero signals with a probability 0.3. We sampled such "negative" points in two different ways. In the first way, we uniformly sampled 30 points from an axis-aligned rectangular region around the microphone array

whose sides are 6 m away from the closest microphone. Then, we discarded all points that are too close to any speaker and obtained a list of candidate points. Finally, we sampled a single target point from the remaining candidates. To ensure that we sample challenging samples frequently, the probability of choosing a candidate point was inversely proportional to the L1-norm to the nearest speaker in the TDoA space. The L1-norm between two vectors is the sum of element-wise absolute differences between the two. The second way we chose negative samples was by running the SRP-PHAT pruning algorithm on the mixture and sampling one of the predicted points with no close speakers. We chose the points that correspond to non-zero target output (positive points) by sampling a target speaker uniformly at random, and shifting the microphone channels to that speaker. For synthetic samples, we directly computed the number of samples to shift each channel by from the speaker positions. For real world samples, since the real world position may be inaccurate, the sample shifts at each channel were computed by running the SRP-PHAT algorithm on the signal from each isolated speaker.

The localization by separation network was trained in two stages. In the first stage, we used a loss function based on the signal-to-noise-ratio (SNR loss) to update the network parameters for positive samples, and we only sample negative points using the first method. This is defined as follows:

$$L_{SNR} = -10 \log \left(\frac{|\mathbf{s}|^2}{|\mathbf{s} - \hat{\mathbf{s}}|^2} \right) \tag{2}$$

where s is the target signal, and ŝ is the model output. In the second stage, we used the L1-norm between the model output and the target signals as our loss function (L1-loss) to update the network parameters for positive samples, and we chose negative samples using the first method described above with a probability of 0.2, and using the second method with a probability of 0.8. Notice that the SNR loss may be very large when the target signal is zero, as is the case for negative samples. So, for negative samples only, we opted to use an L1-loss to prevent the total loss from being dominated by the negative samples for which the network may wrongly output small non-zero values. For the results shown in Fig. 6, the model was trained for the first stage over a total of 50 epochs. The initial learning rate is set to 3×10^{-4}. After 30 epochs, we halve the learning rate if there is no improvement in the validation loss after 4 epochs. The second stage of training occurs over 20 epochs with an initial learning rate of 3×10^{-4}, and we halve the learning rate if there is no improvement in the validation loss for 3 epochs. For the remaining results, the model is trained on the larger, augmented dataset and so we reduce the number of epochs in the first stage to 25.

To obtain the results in Fig. 6, the separation by localization network is trained for 50 epochs with an initial learning rate of 3×10^{-4}. After 30 epochs, we halve the learning rate if there is no improvement in the validation loss for 3 epochs. Once again, we reduced the number of epochs when we trained on the augmented dataset, down to 20 epochs. During training only, we assumed the speaker number is known and set it to the maximum number of concurrent speakers in the dataset. This was done so that we can process the datasets with varying speaker numbers using fixed-size batched tensors. For samples with fewer speakers, we padded the data with zeros to match the size of the largest tensor in the dataset. To compute the loss, we used a loss function based on the scale-invariant-signal-to-noise-ratio SI-SDR loss function, between the output signal and the target signal.

This is defined as:

$$L_{SI\_SDR} = -10 \log \left(\frac{|\frac{\hat{\mathbf{s}}^{\mathbf{T}}\mathbf{s}}{|\mathbf{s}|^2}\mathbf{s}|^2}{|\frac{\hat{\mathbf{s}}^{\mathbf{T}}\mathbf{s}}{|\mathbf{s}|^2}\mathbf{s} - \hat{\mathbf{s}}|^2} \right) \tag{3}$$

where s and $\hat{s}$ are the target signal and model output respectively. We disregarded added targets due to padding or perturbation when computing the loss.

During all training runs, we apply pink and white noise to the mixture signals for data augmentation. For models trained on combined synthetic and real world data, we also compressed and decompressed the audio with a probability of 0.7 to simulate the effects of the lossy codec used by the robot swarm. Since recovering the losses introduced by the codec is not our goal, we applied this distortion to both the mixture signals received at the microphones as well as the target output signals. During training, we perturbed the non-reference mixture channels for synthetic positive examples by time shifting them by up to W samples. While training the separation by localization network, we also randomly dropped one of the speakers in the mixture and/or randomly added a false speaker estimate at a negative position with probability 0.9 to simulate some errors in the localization stage.

For our comparisons with prior speech separation networks, we use SpeechBrain (*3*) implementations of SepFormer and Conv-TasNet, modified to take multiple input channels by increasing the input dimension of the encoder stage to take 7 channels as opposed to one. We use Conv-TasNet with N=512, B=128, H=512, ,P=3, X=8 and R=3. We use SepFormer with the same parameters as the best performing model in (*4*) (without dynamic mixing). We train these networks on a synthetic dataset with similar configurations as our synthetic dataset, but we fix the speaker number to 2. Since the original models operate on 8 kHz audio, while our dataset uses 48 kHz, we multiply the encoder kernel size of both models by a factor of 6, i.e., we increase the encoder kernel size from 16 to 96. For fairness, we re-train our models on this 2 speaker dataset as well to report the results in Fig. 6E.

For our models, we used gradient clipping to clip the gradient L2-norm to 1 and used the Adam optimizer for training. The blind speech separation models we compare with (ConvTasNet and SepFormer), as well as our proposed networks are trained with a batch size of 8. Whenever we evaluated a model, we used the weights from the epoch that yielded the smallest validation loss.

The results reported with a co-located array are from a network trained and evaluated on a modified version of the synthetic 2–5 speaker dataset, where we swap out the distributed microphone array with a co-located microphone array.

Weights rescaling and input normalization. When initializing a model for the first time, we rescaled the weights of all convolutional layers with a reference set to 0.1, as was done in (*5*). Additionally, whenever we run a forward pass with our models, we pre- and post-processed the signal in two ways. First, following prior work (*6*), we normalized the mixture signals so that the entire multi-channel input had zero mean and a standard deviation of 1. We then de-normalized the model output before computing the loss. Second, we left padded the model input with zeros so that the input length was divisible by the product of the stride at each layer of the U-Net. We removed this additional padding at the end of the forward pass.

Runtime evaluation. We ran our pipeline on the GPU machine with 32 CPUs (Intel Xeon Gold 6230), 256 GB memory and 8 GPUs(Nvidia Quadro RTX 6000). Our pipeline can be decomposed

into 4 steps: SRP-PHAT, source localization, clustering, and joint source separation. In the experiment, we test both a large and small network. Both networks had the same architecture but a different parameter number. Specifically, the small network had 4 encoder and 4 decoder stages, with stride length 2, 2, 4 and 4. We also used a smaller initial number of channels (32 channels), and use 1024 features for the mask. Additionally, we reduced the number of residual layers in each encoder and decoder block to 2, and used a kernel size of 5 and a dilation factor of 5 for the residual layers. We ran the pipeline on the 2-speaker synthetic dataset over 1000 3-second samples.

Supplementary Algorithm 1. Estimating arrival time using two microphones

Input:
audio signals from two microphones: y_1, y_2
reference chirp: x
distances between the microphones: d
Output: Arrival time of chirp t_{recv}

$Y_1 \leftarrow$ FFT(y_1)
$Y_2 \leftarrow$ FFT(y_2)
$X \leftarrow$ FFT(x)
$h_1 \leftarrow$ IFFT $(Y_1 \cdot X^H)$
$h_2 \leftarrow$ IFFT $(Y_2 \cdot X^H)$
$N \leftarrow$ Length(h_1)
for $n \leftarrow 1$ to N **do**
  if $\text{IsPeak}(h_1[n])\ \ \&\ \ |h_1(n)| > \text{Thresh}_{\text{noise}}\ \ \&\ \ \frac{\|h_1[n-w:n]\|}{\|h_1[n-2w:n-w]\|} \geq \text{Thresh}_{\text{slope}}$ **then**
    $n_1 \leftarrow n$
    BREAK ▷ w is a fixed window size we set to 8
  end if
end for
for $n \leftarrow 1$ to N **do**
  if $\text{IsPeak}(h_2[n])\ \ \&\ \ |h_2(n)| > \text{Thresh}_{\text{noise}}\ \ \&\ \ \frac{\|h_2[n-w:n]\|}{\|h_2[n-2w:n-w]\|} \geq \text{Thresh}_{\text{slope}}$ **then**
    $n_2 \leftarrow n$
    BREAK
  end if
end for
if $|n_1 - n_2| < d/c * f_s$ **then**
  $t_{recv} \leftarrow (n_1 + n_2)/(2f_s)$
else
  $t_{recv} \leftarrow \min\{n_1, n_2\}/f_s$
end if

Supplementary Algorithm 2. Mapping 3D space to TDoA space

Input:
region of interest in 3D space: RoI
hypercube width W
Microphones positions $\{\mathbf{mic_i}\}_{i=1}^{M}$,
Output: A set of hypercubes $\mathcal{P}$

```
P ← ϕ
VisitedMap(q) ← 0, ∀q ∈ RoI
for q ← RoI By (5, 5, 10) cm do
    if VisitedMap(q) = 1 then
        CONTINUE
    end if
    p ← Calculate_TDoA(q, {mic_i}_{i=1}^M)    ▷ calculate the TDoA values with mic1 as the reference
    P̂ ← ⌊p/W⌉                                 ▷ Division and rounding are element-wise
    P ← P ⋃ P̂
    VisitedMap(q) ← 1
    C_open ← {q}
    C_close ← ϕ
    while C_open ≠ ϕ do
        q′ ← POP(C_open)                      ▷ POP removes and returns the first item from the set
        C_close ← C_close ⋃ q′
        for q̄ ← Neighbor3D(q′) do             ▷ Neighbor3D returns the neighbor points in 3D space
            P̄ ← Calculate_TDoA(q̄, {mic_i}_{i=1}^M)
            if ‖P̂ − P̄‖_∞ ≤ W then
                C_open ← C_open ⋃ q̄
                VisitedMap(q̄) ← 1
            end if
        end for
    end while
end for
```

Supplementary Algorithm 3. Joint 2D localization and separation

Input:

Microphones positions $\{\mathbf{mic_i}\}_{i=1}^{M}$
Mixture input $x \in \mathbb{R}^{M \times T}$
region of interest in 3D space: RoI

Output: 2D localization of speakers $\mathcal{S}$ and their separation results $\mathcal{Y}$

$\mathcal{S} \leftarrow \phi$
$\mathcal{Y} \leftarrow \phi$
$\mathcal{P} \leftarrow \mathrm{Map\_3D\_TDoA}(whole\_3D\_space)$
$\mathcal{P}_{pruned} \leftarrow \mathrm{SRP\_Pruning}(\{\mathbf{mic_i}\}_{i=1}^{M}, x, \mathcal{P})$ ▷ SRP-PHAT based pruning
$\mathcal{S} \leftarrow \mathrm{Clustering}(\mathcal{P}_{pruned}, \{\mathbf{mic_i}\}_{i=1}^{M}, x)$
$X \leftarrow \phi$
for $s \in \mathcal{S}$ **do**
  $x' \leftarrow$ SHIFT(x, s)
  $X \leftarrow X \bigcup x'$
end for
$\mathcal{Y} \leftarrow \mathrm{separation\_net2}(X)$ ▷ Network in Fig. 5C

Supplementary Algorithm 4. Clustering

Input: Hypercubes sets from pruning with width=4, $\mathcal{P}_{W4}$,
  Microphones positions $\{\mathbf{mic_i}\}_{i=1}^{M}$,
  Mixture input $x \in \mathbb{R}^{M \times T}$
Output: Speakers' 2D localization results $\mathcal{S}$
  $\mathcal{S} \leftarrow \phi$
  $\mathcal{P}_{W2}, Y \leftarrow \text{Separation\_For\_Localization}(\mathcal{P}_{W4}, \{\mathbf{mic_i}\}_{i=1}^{M})$
  $Y_{sorted}, \mathcal{P}_{sorted} = \text{SORT}(Y,\ P_{W2},$ key $= \{\|y\|,\ \forall y \in Y\})$ ▷ Sort the list by the keys values
  $C \leftarrow \phi$
  for $i \in$ [0, Size(Y_{sorted})-1] **do**
    $y \leftarrow Y_{sorted}[i]$
    $p \leftarrow \mathcal{P}_{sorted}[i]$
    $\text{Match\_Flag} \leftarrow \text{False}$
    for $c \in C$ **do**
      $y_c, p_c \leftarrow c[0]$
      if $\|p - p_c\|_\infty \leq 4\ \ \&\ \ \text{SI}-\text{SDR}(y, y_c) > \text{Thresh}_{\text{sim}}$ **then** ▷ we set $\text{Thresh}_{\text{sim}} = -4$
        $c \leftarrow c \bigcup (y, p)$
        Match_Flag ← True
        BREAK
      end if
    end for
    if $\text{Match\_Flag} = \text{False}\ \ \&\ \ \neg\ \text{Segment\_wise\_Sim}(y, C)$ **then**
      $c \leftarrow \{(y, p)\}$
      $C \leftarrow C \bigcup c$
    end if
  end for
  for $c \in C$ **do**
    p ← $\text{Weighted\_Sum}(c)$ ▷ Signal powers are used as the weights
    s = Map_TDoA_3D(p) ▷ Map the hypercube p from TDoA space to the 3D space
    $\mathcal{S} \leftarrow \mathcal{S} \bigcup s$
  end for

Supplementary Algorithm 5. Separation_For_Localization

Input: Hypercubes sets from pruning with width=4: $\mathcal{P}_{W4}$,
Microphones positions $\{\mathbf{mic_i}\}_{i=1}^{M}$,
Mixture input $x \in \mathbb{R}^{M \times T}$
Output: Hypercube sets after division with width=2: $\mathcal{P}_{W2}$, their separation output Y

$\mathcal{P}_{W2} \leftarrow \phi$
for $p \in \mathcal{P}_{W4}$ **do**
  $x' \leftarrow \mathrm{SHIFT}(x, p)$
  $y \leftarrow \mathrm{Separation\_net1}(x'|w = 4)$ ▷ Network in Fig. 5B
  $d \leftarrow \|\mathbf{mic_1} - \mathrm{Map\_TDoA\_3D}(p)\|$ ▷ Mapping from TDoA space to the 3D space
  if $\mathrm{Max\_Win\_Power}(y) > \frac{0.01}{1+d}$ **then** ▷ Maximum power within a 0.25 s window
    $\mathcal{P}_2 \leftarrow \mathrm{Divide4to2}(p)$ ▷ Divide the 4-width hypercube into 2-width hypercubes
    $\mathcal{P}_{W2} \leftarrow \mathcal{P}_{W2} \bigcup \mathcal{P}_2$
  end if
end for
$Y \leftarrow \phi$
$\mathcal{P} \leftarrow \phi$
for $p \in \mathcal{P}_{W2}$ **do**
  $x' \leftarrow \mathrm{SHIFT}(x, p)$ ▷ shift the multi-channel audios x based on p
  $y \leftarrow \mathrm{Separation\_net1}(x'|w = 2)$ ▷ Network in Fig. 5B
  $d \leftarrow \|\mathbf{mic_1} - \mathrm{Map\_TDoA\_3D}(p)\|$ ▷ Mapping from TDoA space to the 3D space
  if $\mathrm{Max\_Win\_Power}(y) > \frac{0.01}{1+d}$ **then** ▷ Maximum power within a 0.25 s window
    $Y \leftarrow Y \bigcup y$
    $\mathcal{P} \leftarrow \mathcal{P} \bigcup p$
  end if
end for
$\mathcal{P}_{W2} \leftarrow \mathcal{P}$

Supplementary Algorithm 6. Segment_wise_Sim

Input: input signal y,
source sets C,
Output: True/False whether the input signal y is a phantom source from the sets of source C

$\text{Idx} \leftarrow \text{Split}(y)$
▷ Split into non-silent intervals with max length of 8000 samples, return their start/end index
$\text{Sim}_{\text{seg}} \leftarrow \phi$
for $(i_{start}, i_{end}) \in \text{Idx}$ **do**
  $\text{Sims} \leftarrow \phi$
  for $c \in C$ **do**
    $y_c, p_c \leftarrow c[0]$
    $s \leftarrow$ Time-Invariant-SI-SDR$(y[i_{start} : i_{end}], y_c[i_{start} : i_{end}])$
    $\text{Sims} \leftarrow \text{Sims} \bigcup s$
  end for
  $\text{Sim}_{\text{seg}} \leftarrow \text{Sim}_{\text{seg}} \bigcup \max(\text{Sims})$
end for
if $\max(\text{Sim}_{\text{seg}}) > \text{Thresh}_{\text{sim1}}$ & $\min(\text{Sim}_{\text{seg}}) > \text{Thresh}_{\text{sim2}}$ **then**
  RETURN True ▷ we set $\text{Thresh}_{\text{sim1}} = -1$ and $\text{Thresh}_{\text{sim2}} = -6$
else
  RETURN False
end if

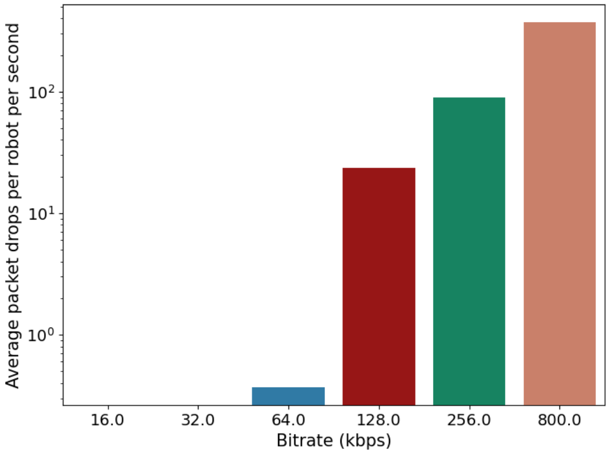

Supplementary Figure 1: **Bluetooth packet loss in our swarm versus compression.** At higher audio compression ratios, the bit rate required by each swarm robot reduces and as a result the packet drops per robot also decrease.

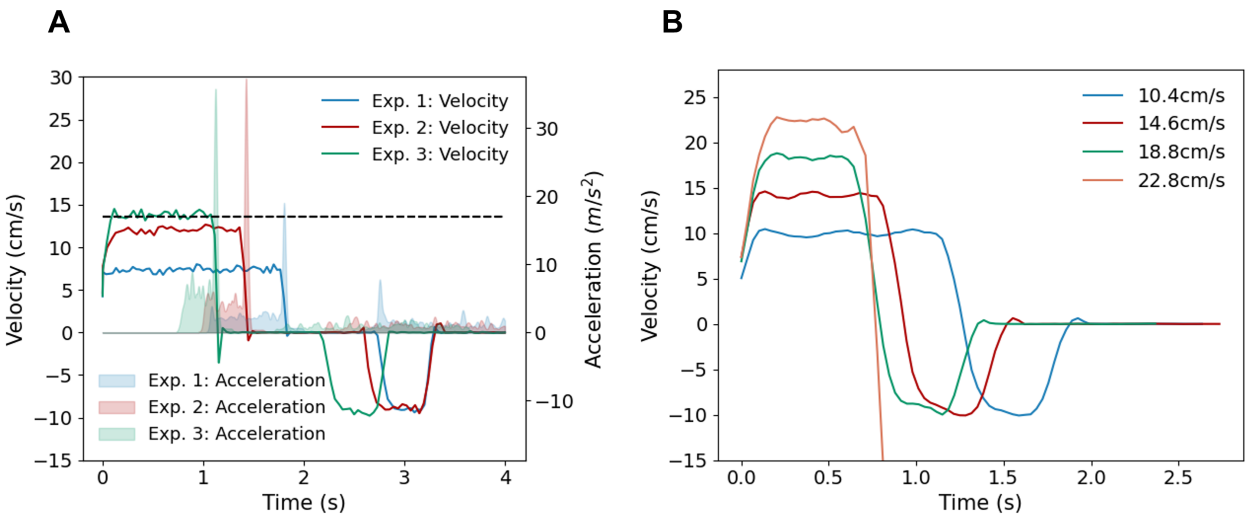

Supplementary Figure 2: **Detecting collisions with objects and surface edges.** The plots show the velocity measurements with a moving average of 5 samples. The acceleration measurements are computed with a Gaussian kernel with a standard deviation of 10 while maintaining the maximum amplitude. At higher speeds, the accelerometer registers larger spikes. At lower speeds, the robot has more time to react to edges without falling off. After detecting a collision or an edge, the robot moves backward slightly.

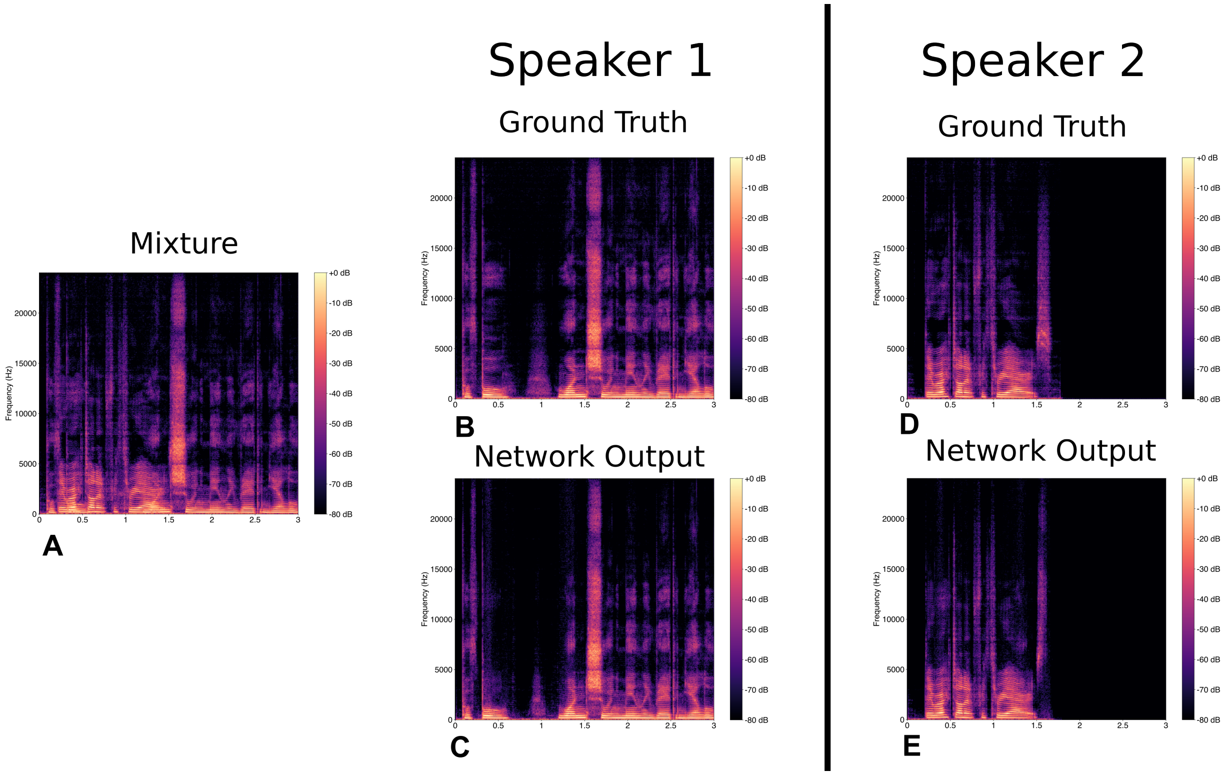

Supplementary Figure 3: **The spectrograms corresponding to the waveforms shown in Figure 6A.** The mixture signal is shown in **A)**. We show the ground truth audio for the first speaker in **B)** and the corresponding network output audio in **C)**. Likewise, the spectrograms for the ground truth audio of the second speaer is shown in **D)** and the network output for this speaker is shown in **E)**.

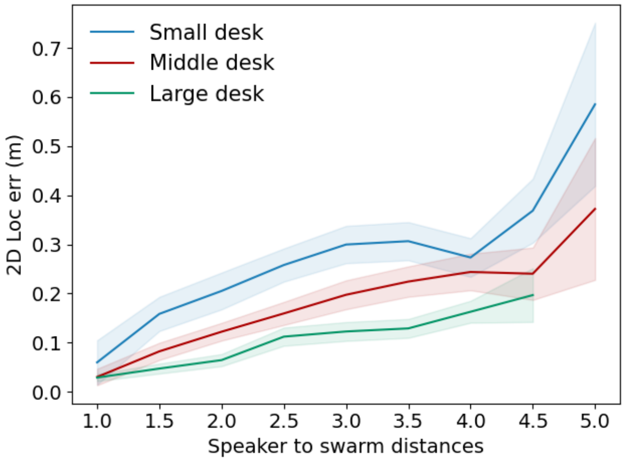

Supplementary Figure 4: **Speaker 2D localization error versus desk size.** When our acoustic swarm spread out on a smaller desk, they have a smaller aperture. At farther distances, a smaller aperture size increases the localization error. (The error bar is the 90% Confidence Intervals using the t-distribution)

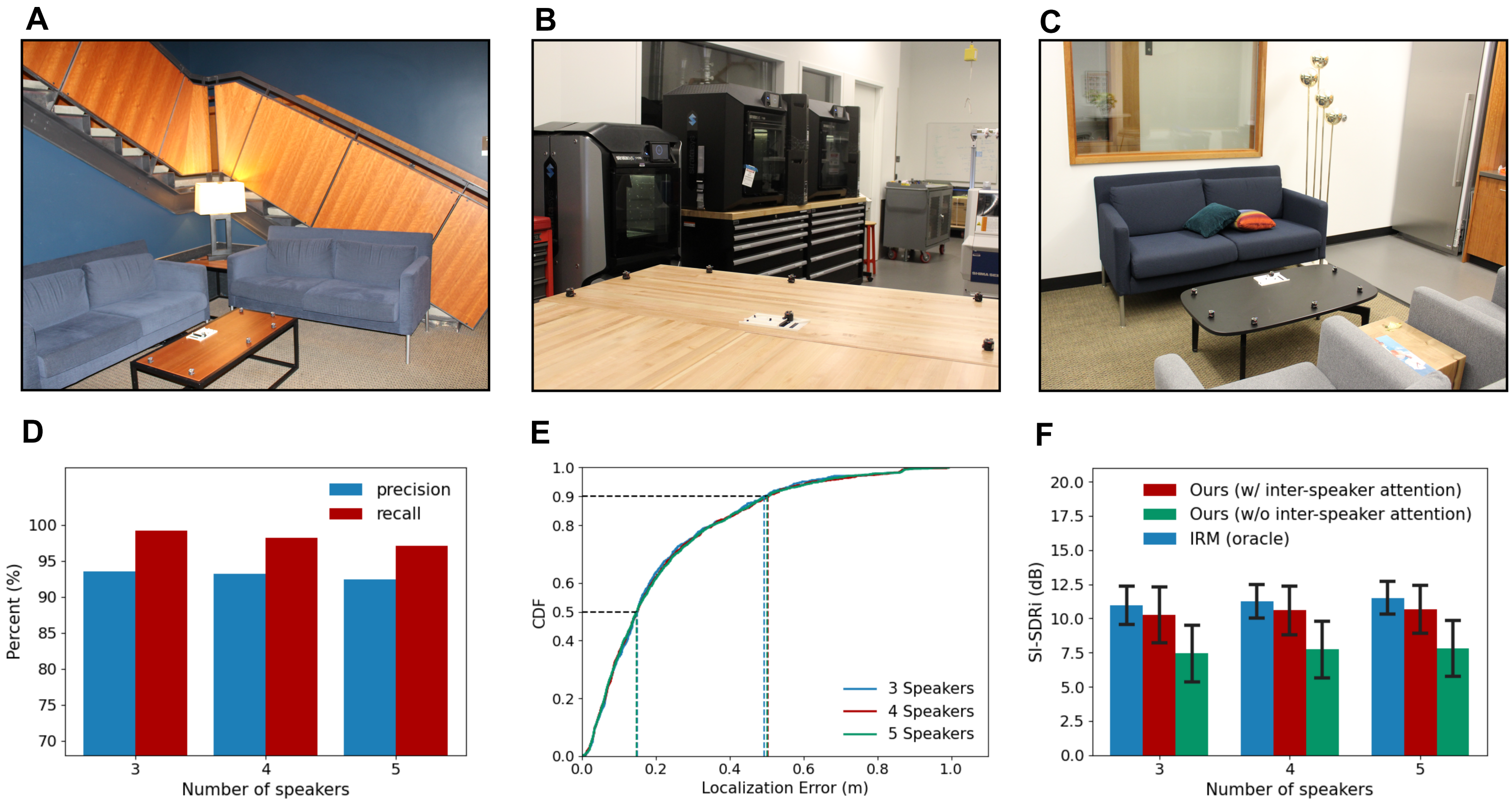

Supplementary Figure 5: **Real-world evaluation.** **A-C)** show three of the five previously unseen real-world reverberant environments that were used for testing our system. **D)** shows the precision and recall of our system for correctly identifying the speech signals as a function of different number of concurrent speakers. **E)** plots the cumulative distribution function (CDF) for the 2D localization errors for different number of speakers across all the tested environments. **F)** compares our approach with and without our attention-mechanism with an oracle approach (IRM) (the error bar shows the standard deviation).

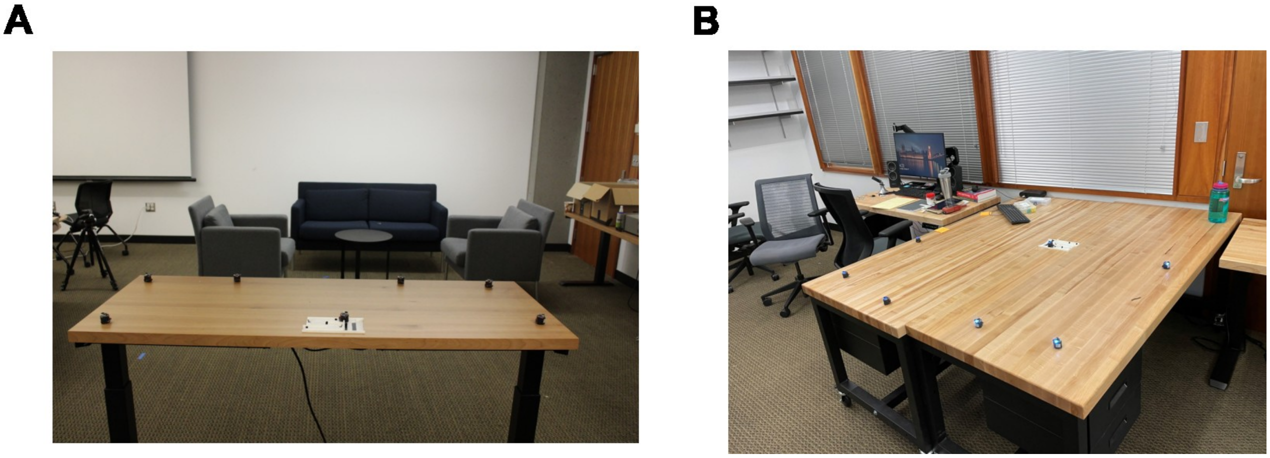

Supplementary Figure 6: **Additional real-world testing environments. A)** and **B)** show the remaining two testing environments that are used in our real-world evaluation.

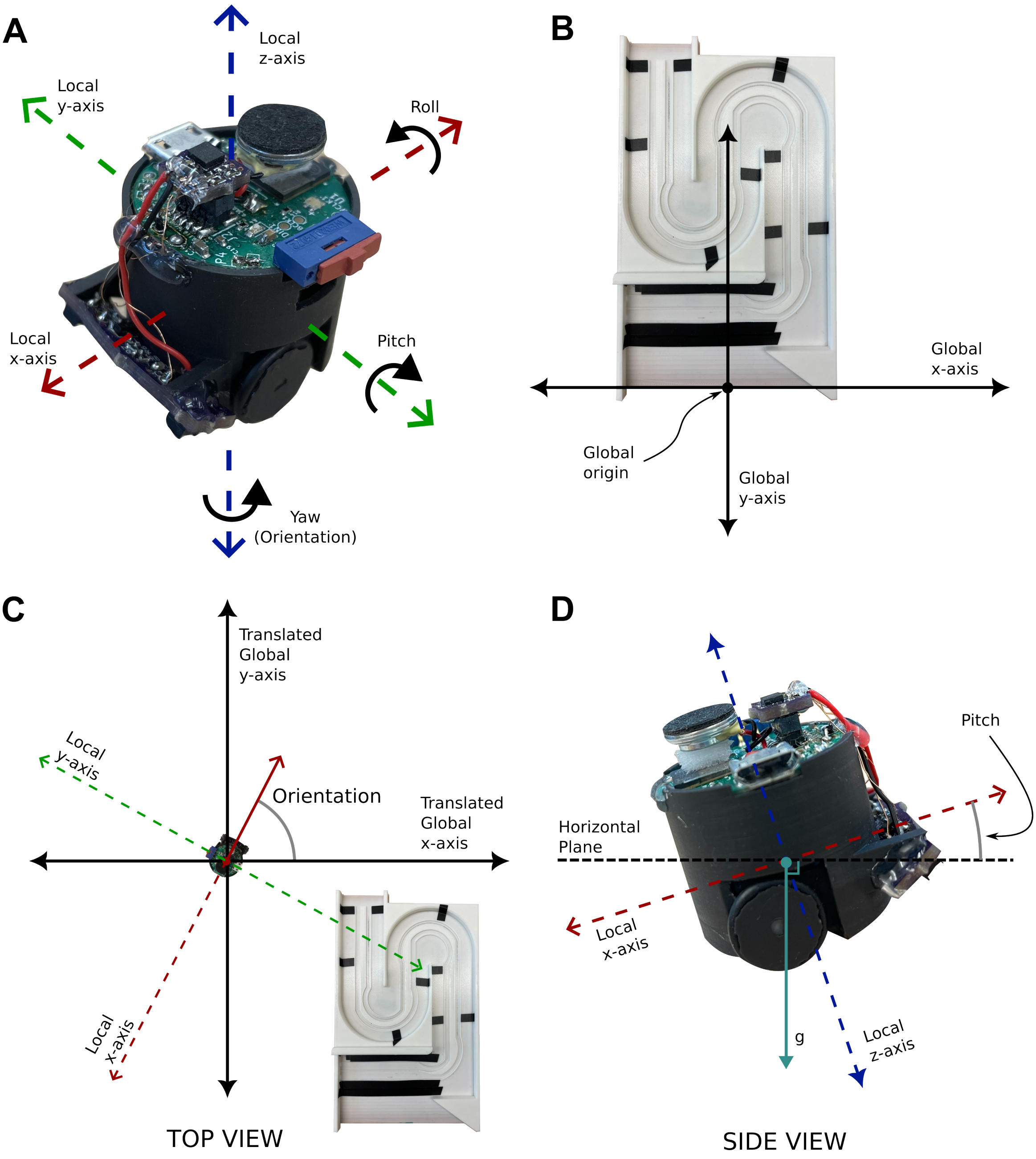

Supplementary Figure 7: **Robot local reference frame and swarm global reference frame. A)** A robot's XYZ-axes according to its own reference frame. **B)** The global reference frame as defined according to the base station. **C)** The definition of a robot's orientation with respect to the axes of a translated global reference, centered at the robot. **D)** The robot's pitch as defined relative to gravity.

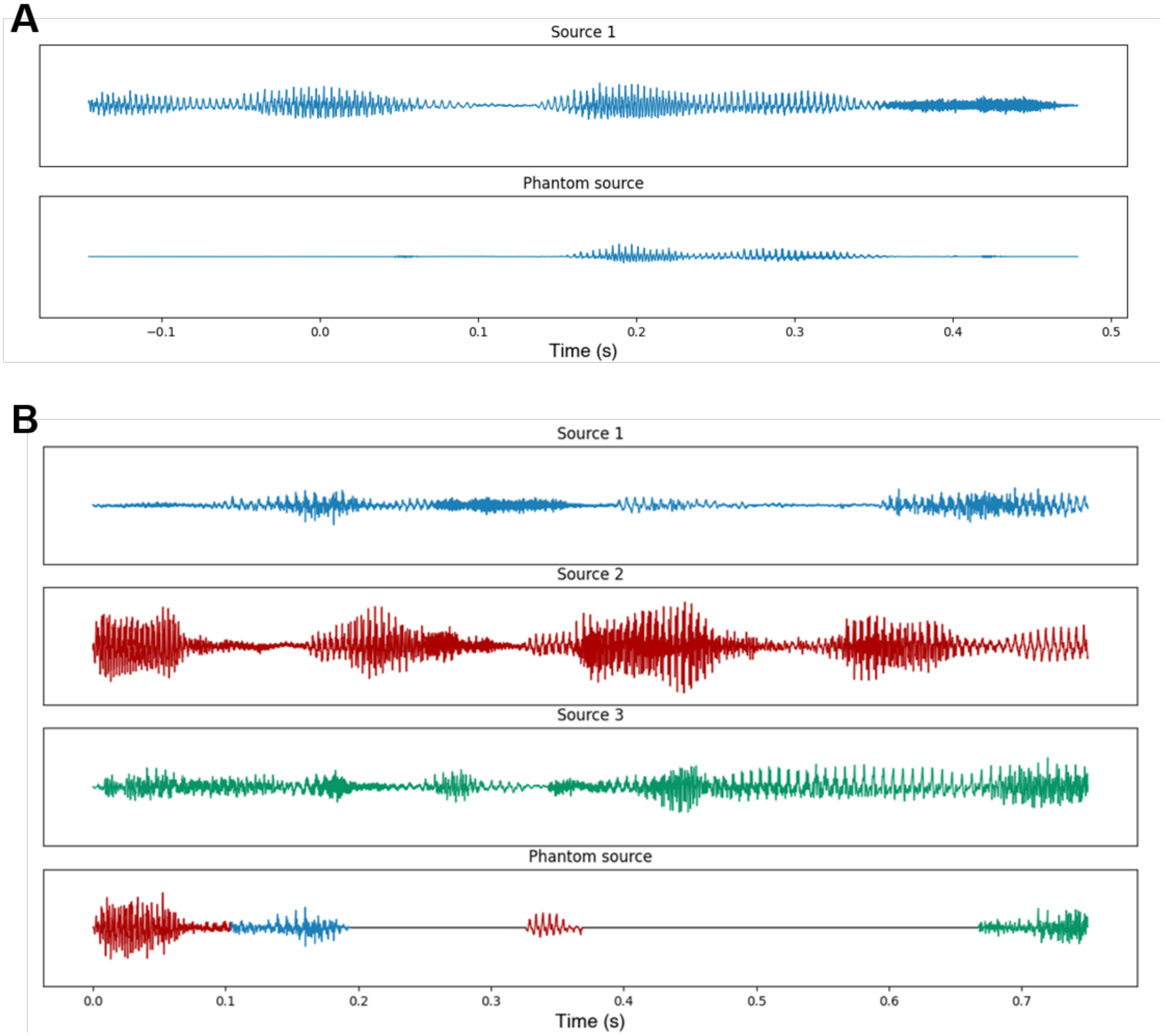

Supplementary Figure 8: **Examples of phantom speakers.** There are two types of phantom sources. The first kind shown in **A)** are close to the actual source location but has parts of the source while being attenuated. The second kind shown in **B)** are phantom sources which are combinations of multiple sources due to reverberations.

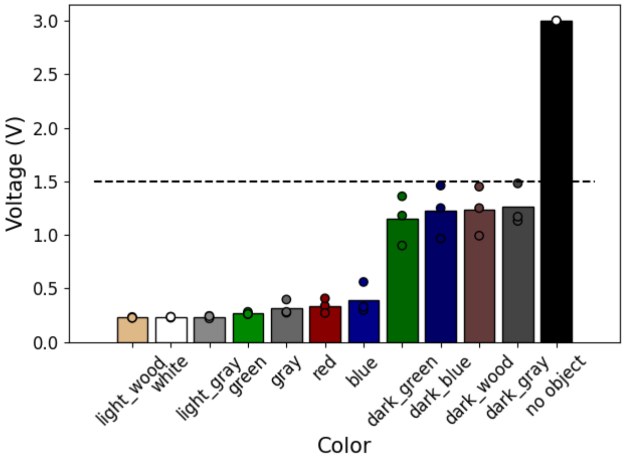

Supplementary Figure 9: **Output voltage of the photointerruptors for different color tables.** Darker table colors result in higher voltage. At the edge of a table (no surface) the voltage is much higher. We use the dashed line as our threshold to detect surface edges.

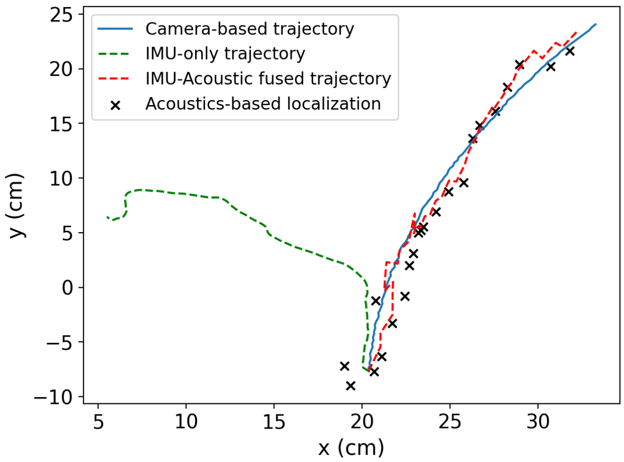

Supplementary Figure 10: **Sensor coordination experiment for robot localization.** The blue curve is the actual robot trajectory recorded by the top camera. The green line is the robot trajectory inferred from only IMU data. The black cross symbols are the localization results from the acoustic data. The red curve is the trajectory inferred by our fusion algorithm, using $w_P^I = w_P^A = 0.5$.

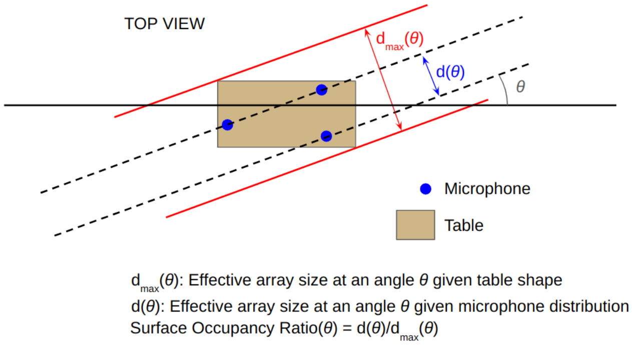

Supplementary Figure 11: **Surface Occupancy Ratio.** This metric compares the apparent array size as seen at a particular angle to the maximal array size that can be achieved with the surface used.

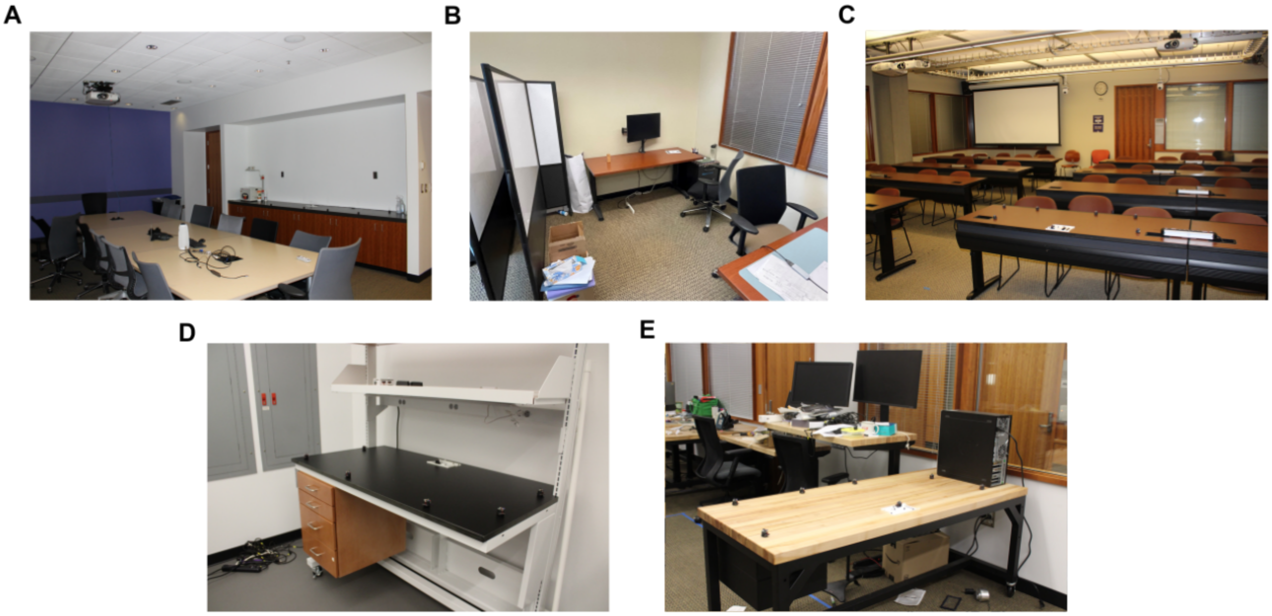

Supplementary Figure 12: **Real-world training and validation environments. A-D)** show the four training environments that are used to train our models and **E)** shows the environment used for validation.

Mic pos err	0	1cm	2cm	3cm	4cm
Precision	97.4%	95.6%	95.8%	94.0%	92.6%
Recall	96.1%	95.8%	94.1%	91.0%	88.0%
Median 2D loc err	0.104	0.123	0.152	0.175	0.258
90% 2D loc err	0.345	0.395	0.514	0.654	0.79
SI-SDRi (dB)	17.7	17.8	17.6	17.5	18.4

Supplementary Table 1: Effect of microphone position errors on 2D localization and speech separation.

RT60 (s)	<0.2	0.2-0.4	0.4-0.6	> 0.6
Precision	99.3%	98.6%	95.8%	93.0%
Recall	97.3%	95.3%	93.3%	88.4%
Median 2D loc err (m)	0.044	0.046	0.048	0.047
90% 2D loc err	0.122	0.130	0.170	0.176
SI-SDRi (dB)	18.23	14.1	11.34	9.68

Supplementary Table 2: Performance with different reverberation times (RT60).

Room	Purpose	Dataset	Size (meters)	RT60 (s)
1	Conference Room	Train	9x6x3	0.69+/-0.04
2	Office	Train	6x6x3	0.42+/-0.04
3	Classroom	Train	9x9x3	0.50+/-0.02
4	Laboratory	Train	10x5x5	0.78+/-0.07
5	Office	Validation	10x5x3	0.47+/-0.02
6	Living Room	Test	5x5x6	0.72+/-0.03
7	Laboratory	Test	10x10x3	0.41+/-0.01
8	Kitchen	Test	5x5x3	0.49+/-0.04
9	Office	Test	10x5x3	0.46+/-0.02
10	Office with Living Area	Test	9x9x3	0.50+/-0.05

Supplementary Table 3: **Description of rooms used for real world evaluation.** Rooms used in our different dataset splits, as well as their corresponding sizes and approximate reverberation times.

Model configuration	SI-SDRi (dB)
Stride = 256, Channel = 64, Feature = 4096	12.83
Stride = 64, Channel = 64, Feature = 4096	**17.14**
Stride = 64, Channel = 32, Feature = 4096	16.34
Stride = 64, Channel = 64, Feature = 2048	*17.05*

Supplementary Table 4: **Ablation study for our separation using localization mode.** The stride size shown is the product of the stride sizes across the U-Net layers. The channel is the number of channels at the first layer of the U-Net. The feature is the number of latent representations on the reference channel.

Supplementary references

1. Scheibler, Robin *et al.* Pyroomacoustics: A python package for audio room simulation and array processing algorithms *IEEE international conference on acoustics, speech and signal processing (ICASSP)* (2018).

2. Sainburg, Tim *et al.* Finding, visualizing, and quantifying latent structure across diverse animal vocal repertoires *PLoS computational biology* (2020).

3. Ravanelli, Mirco *et al.* SpeechBrain: A General-Purpose Speech Toolkit *arxiv* (2021).

4. Subakan, Cem *et al.* Attention is all you need in speech separation *IEEE International Conference on Acoustics, Speech and Signal Processing (ICASSP)* (2021).

5. Défossez, Alexandre *et al.* Demucs: Deep Extractor for Music Sources with extra unlabeled data remixed *arxiv* (2019).

6. Jenrungrot, Teerapat *et al.* The cone of silence: Speech separation by localization *Advances in Neural Information Processing Systems* (2020).
