# Supplementary material for: Creating speech zones with self-distributing acoustic swarms
Source: Nat Commun. 2023 Sep 21;14:5684. doi: 10.1038/s41467-023-40869-8 (PMC10514314; doi:10.1038/s41467-023-40869-8)
Supplement: Supplementary file 3 — Description of Additional Supplementary Files [file 41467_2023_40869_MOESM3_ESM.pdf]

Description of Additional Supplementary Files

File Name: Supplementary Movie 1
Description: Creating conversational zones. The acoustic swarm on the table is used to separate the two conversations into two different conversational zones by using the separated speech and the estimated 2D locations of the speakers. Speakers utter dialogues from the DailyDialog dataset

File Name: Supplementary Movie 2
Description: 2D localization of multiple moving speakers. The video shows the 2D locations estimated by our system for both the concurrent speakers walking around the room. The acoustic swarm is spread out across the table in the background. Speakers read book passages from Project Gutenberg.

File Name: Supplementary Movie 3
Description: Creating quiet and active zones. The acoustic swarm on the table is used to create both active and quiet zones. Active zones are the only 2D regions in the room where speech can be extracted from and quiet zones are the 2D regions in the room where speech is suppressed. The input mixture was denoised by capturing 500~ms of environment noise before running the algorithm.

File Name: Supplementary Movie 4
Description: Swarm 2D localization. The video shows the robot moving within the base and computing distances from each of the “virtual landmarks” to the remaining robots. We then show the predicted 2D positions as estimated by our system.

File Name: Supplementary Movie 5
Description: Swarm dispersal. The video shows the various steps involved in dispersing the swarm across the table. The robots first discover their order within the base and then arrange themselves on the landmarks within the base. The robots then move along equally separated directions across the surface.

File Name: Supplementary Movie 6
Description: Swarm dispersal with objects. The video shows dispersal in the presence of objects spread out across the table. The robots use their IMU readings to detect collisions and move away from the colliding objects.

File Name: Supplementary Movie 7
Description: Swarm returning to the base. The video shows various steps involved in returning all the robots back to the base. The swarm first moves towards the base and then one after the other dock with the base station.

File Name: Supplementary Movie 8

Description: Synthetic data example results. The video shows synthetic data examples of our 2D localization and speech separation with 3, 4 and 5 concurrent speakers in a room.

File Name: Supplementary Movie 9
Description: Real-world example results. The video shows samples of our 2D localization and speech separation with 3, 4 and 5 concurrent speakers in three different previously unseen real-world reverberant rooms.
